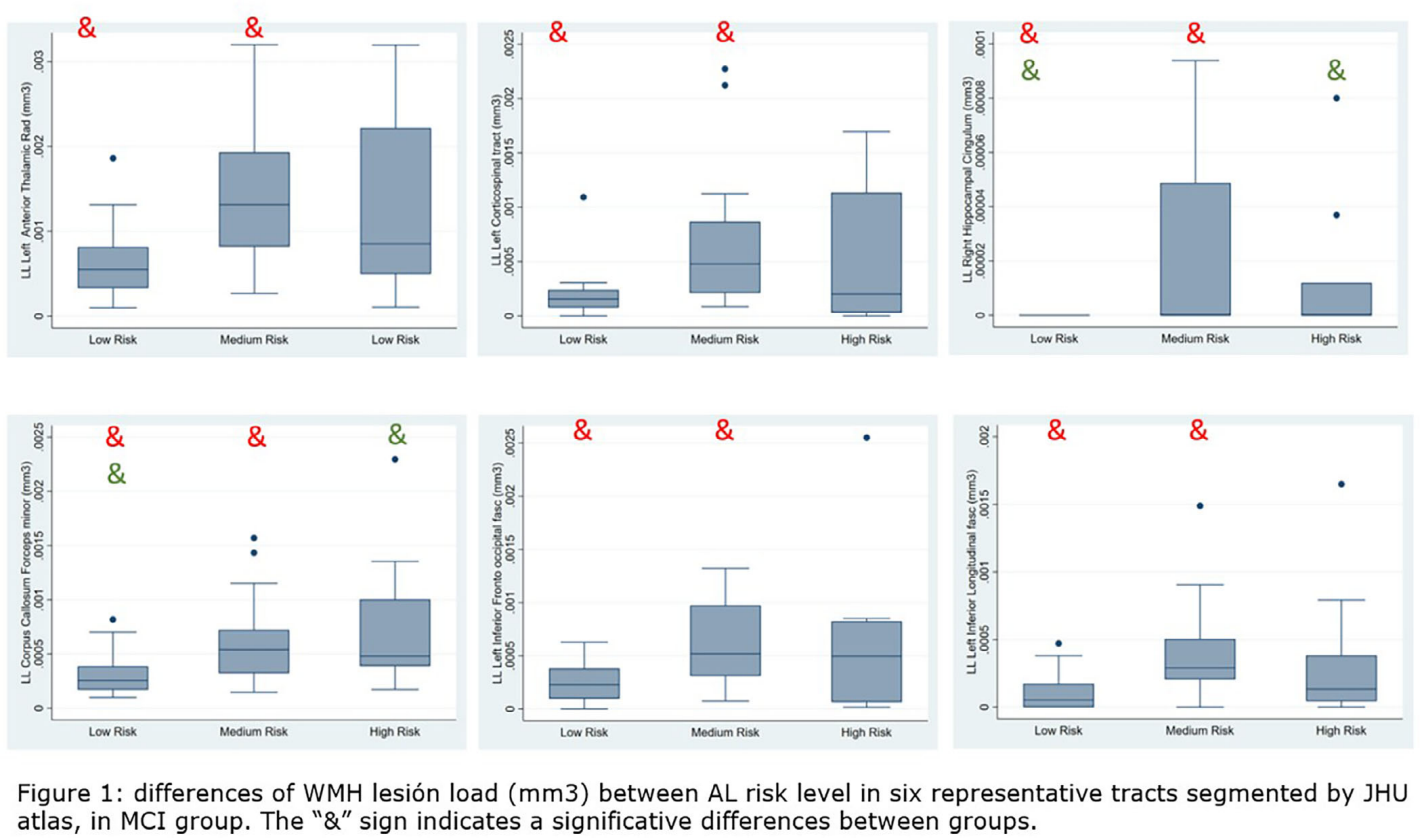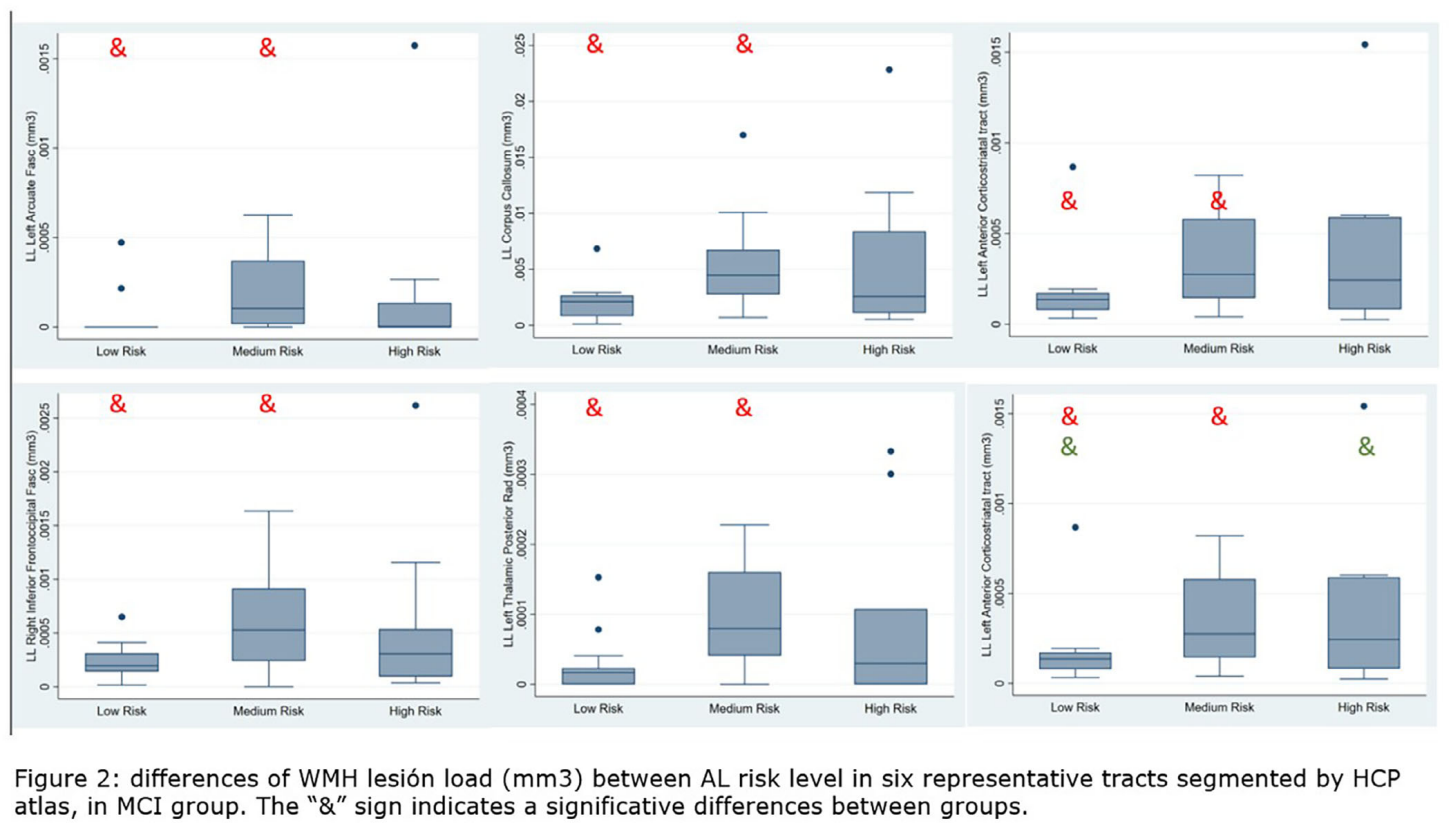# Differences between white matter hyperintensities lesion load across allostatic load risk in cognitive complaint subjects

**DOI:** 10.1002/alz.084760

**Published:** 2025-01-09

**Authors:** Patricio Riquelme Contreras, Fernando Henriquez, Cecilia Gonzalez Campo, Ingrid Buller‐Peralta, Daniela Thumala, Patricia Lillo, Cecilia Okuma, Michele Demanet, María Francisca Damm, Claudio Román Godoy, Pamela Guevara Alvez, El‐Deredy Wael, Graciela Muniz‐Terrera, Andrea Slachevsky Chonchol

**Affiliations:** ^1^ Department of Medical Technology. Faculty of Medicine. Universidad de Chile, Santiago Chile; ^2^ Masters in biological sciences / Neurosciences. Universidad de Valparaíso, Chile., Valparaíso Chile; ^3^ Geroscience Center for Brain Health and Metabolism (GERO), Santiago Chile; ^4^ Neuropsychology and Clinical Neuroscience Laboratory (LANNEC), Physiopathology Department ‐ ICBM, Neuroscience and East Neuroscience Departments, Faculty of Medicine, Universidad de Chile, Santiago Chile; ^5^ Memory and Neuropsychiatric Center (CMYN), Neurology Department, Hospital del Salvador and Faculty of Medicine, Universidad de Chile, Santiago Chile; ^6^ Interdisciplinary Center for Neuroscience (NeuroUC) ‐ Laboratory for Cognitive and Evolutionary Neuroscience ‐ Medicine School ‐ Pontificia Universidad Católica de Chile, Santiago Chile; ^7^ Memory and Neuropsychiatric Center (CMYN), Neurology Service, Hospital del Salvador and Faculty of Medicine, Universidad de Chile, Santiago Chile; ^8^ Universidad de Chile, Santiago Chile; ^9^ CONICET, Buenos Aires Argentina; ^10^ Cognitive Neuroscience Center (CNC), Universidad de San Andrés, Buenos Aires, Buenos Aires Argentina; ^11^ University of Edinburgh, Edinburgh, Edinburgh UK; ^12^ Laboratory of Neuropsychology and Clinical Neuroscience (LANNEC), Physiopathology Program‐ICBM, East Neurologic and Neurosciences Departments, Faculty of Medicine, University of Chile, Santiago Chile; ^13^ Department of Psychology, University of Chile, Santiago Chile; ^14^ Universidad de Chile, Santiago, Santiago Chile; ^15^ Instituto de Neurocirugía Dr. Alfonso Asenjo, Santiago Chile; ^16^ Geroscience center for mental health and metabolism (GERO), Santiago de Chile, Metropolitana Chile; ^17^ Memory and Neuropsychiatry Clinic (CMYN), Santiago de Chile, Metropolitana Chile; ^18^ Health Engineering I&D Center. Universidad de Valparaíso., Valparaíso, Valparaíso Chile; ^19^ Geroscience center for mental health and metabolism, Santiago de Chile, Metropolitana Chile; ^20^ Biomedical Engineering Department. Universidad de Concepción, Concepción, Bio Bio Chile; ^21^ Faculty of Engineering. Universidad de Valparaíso., Valparaíso, Valparaíso Chile; ^22^ Edinburgh Dementia Prevention, University of Edinburgh, Edinburgh UK; ^23^ Centre for Clinical Brain Sciences at the University of Edinburgh, Edinburgh UK; ^24^ University of Edinburgh, Edinburgh UK

## Abstract

**Background:**

Chronic exposure to stress, quantified by allostatic load (AL), has been postulated as a cause of structural brain changes in the context of dementia. White matter hyperintensities (WMH), detected in MRI FLAIR, are a common brain abnormality representing small vessel disease or degenerative changes in the brain. Here, we studied differences in tract‐specific WMH volume across three risk levels of AL in Chilean subjects with cognitive complaint, to explore links between chronic stress exposure and prodromal steps of dementia.

**Method:**

The study included 135 subjects with cognitive complaint from a chilean cohort. Based in MoCA test, subjects were classified in mild cognitive impairment (MCI) (47) and subjective cognitive complaint (SCC) (88). To measure AL, we employed 15 metabolic, cardiovascular and immunity biomarkers, and the scores were classified in low, medium, and high risk levels of AL. WMH were calculated with Lesion Segmentation Toolbox, in SPM 12, and segmented according to John Hopkins University (JHU) and Human Connectome (HCP) atlases. To explore differences in tract‐specific WMH volume per risk level AL, we used a Kruskal Wallis test in each cognitive group (SCC and MCI). In significant tracts, a U Mann ‐ Whitney test was performed to establish the paired AL risk groups with significance.

**Result:**

In the MCI group, we found a higher tract‐specific WMH load as a function of AL risk level in 10 tracts of the JHU atlas, and in 31 tracts of the HCP atlas. Differences were observed between low and medium‐risk of AL, with medium‐risk group showing a higher WMH load than low‐risk group.

**Conclusion:**

We observed significative differences in tract‐specific WMH lesion between low and medium risk of AL, only in MCI. It is according with literature that show the WMH burden could be associated with cognitive deficit, but not it’s risk. In this study, we further establish a link between chronic stress exposure and WM structural changes in early stages of neurocognitive disorders. We suggest that AL is a global measurement that could mediate the relation between vascular or neurodegenerative factors to WMH origin. Use of neurodegenerative and cardiovascular biomarkers can be useful to explore this mediation.